# Factors associated with condom use with non-commercial partners among sexually-active transgender women in Cambodia: findings from a national survey using respondent-driven sampling

**DOI:** 10.1186/s12889-019-6656-x

**Published:** 2019-03-20

**Authors:** Siyan Yi, Amelia Plant, Sovannary Tuot, Phalkun Mun, Srean Chhim, Navy Chann, Pheak Chhoun, Carinne Brody

**Affiliations:** 1 Saw Swee Hock School of Public Health, National University of Singapore and National University Health System, 12 Science Drive 2, #10-01, Singapore, 117549 Singapore; 2 KHANA Center for Population Health Research, Phnom Penh, Cambodia; 3 Center for Global Health Research, Touro University California, Vallejo, USA; 4 Reproductive Rights and Programming Consultant, Cairo, Egypt; 5 National Center for HIV/AIDS, Dermatology and STD, Phnom Penh, Cambodia; 6 FHI 360, Phnom Penh, Cambodia

**Keywords:** HIV, Condom use, zRisk factors, Non-commercial partners, Transgender women, Cambodia

## Abstract

**Background:**

Globally, the prevalence of HIV among transgender women is much higher than that of the general adult population. This can be explained by the persistently low rate of consistent condom use among this population. This study was therefore conducted to explore factors associated with consistent condom use among sexually-active transgender women in Cambodia, specifically with their non-commercial partners.

**Methods:**

Data used for this study were collected as part of the National Integrated Biological and Behavioral Survey 2016. Participants were recruited from the capital city of Phnom Penh and 12 other provinces with high burden of HIV using the Respondent-Driven Sampling (RDS) method. Face-to-face interviews were conducted using a structured questionnaire. Weighted multivariate logistic regression analysis was conducted to explore independent factors associated with consistent condom use.

**Results:**

This study included 1202 transgender women who reported having anal sex with at least one male partner not in exchange for money or gifts in the past three months. The mean age of the participants was 26.0 (SD = 7.0) years. Of the total, 41.5% reported always using condoms with male non-commercial partners in the past three months. After adjustment, the likelihood of consistent condom use was significantly higher among participants who resided in an urban community (AOR = 1.7, 95% CI = 1.1–2.6), had attained at least 10 years of formal education (AOR = 1.8, 95% CI = 1.2–2.7), perceived that they were likely or very likely to be HIV infected (AOR = 2.9, 95% CI = 2.0–4.1), reported drinking alcohol two to three times per week (AOR = 3.1, 95% CI = 1.1–8.3), reported using amphetamine-type stimulants (AOR = 1.9, 95% = 1.1–3.8) or other drugs (AOR = 7.6, 95% CI = 1.5–39.5), and reported inconsistent condom use with male commercial partners in the past three months (AOR = 4.3, 95% CI = 1.8–10.4) compared to that of their respective reference group.

**Conclusions:**

This study confirms the low rates of condom use, particularly in non-commercial relationship, among transgender women in Cambodia. To address these concerns, efforts towards education about effects of multiple, concurrent relationships, and inconsistent condom use should be reinforced among transgender women.

## Background

Transgender women, individuals who self-identify as female despite being assigned as male at birth, are a vulnerable population globally. They experience a high degree of social marginalization that results in mental health challenges, substance abuse, and exposure to violence—all risk factors for HIV and other sexually transmitted infections (STIs) [1–6]. As a result of these risk factors, they have higher rates of HIV and STIs than cisgender individuals, those whose gender identity corresponds with sex assigned at birth. A 2013 global systematic review found the probability of HIV infection among transgender women to be 48.8 times [95% confidence interval (CI) 21.2–76.3] higher than that in the general adult population [7].

Along with the psychological challenges that frequently lead transgender women to engage in sexual risk behaviors, such as condomless sex, they face societal judgment related to their gender identity. They are often discriminated against when applying for jobs and accessing health services [8, 9]. With the lack of employment opportunities, many transgender women turn to sex work [10]. The double stigmatization of gender identity and prostitution makes this population even less likely to access health services [7, 9, 10]. This lack of access has serious consequences, as the risk of HIV and other STIs is also higher among transgender women who are engaging in transactional sex [10, 11]. Sex workers may be less able to negotiate condom use because of weakened bargaining power, economic instability, low self-esteem, and other psychological problems [6, 9, 12]. Because of stigmatization, many transgender women experience financial isolation or destitution, and have had to make the difficult decision to engage in sex without a condom as an economic survival strategy [5, 10, 13].

Condomless anal intercourse is an important risk factor for HIV and STI transmission [14, 15]. In a 2016 HIV prevalence study among transgender women in Cambodia, respondents who reported not using a condom at last anal intercourse had 3.8 times the odds of HIV infection [16]. Transgender women are more likely to be the receptive partner in a sexual encounter, increasing their risk of HIV and STIs if their rate of condomless anal intercourse is high [2, 8]. Multiple factors have been shown to be correlated with inconsistent condom use among men who have sex with men (MSM) and transgender women globally: HIV risk perception [17], drug and alcohol use [4, 10, 18, 19], depressive symptoms [4, 9, 18, 20, 21], history of abuse [21–23], low levels of education [18], and having sex with a commercial partner [12, 20, 24]. In these studies, condom use is treated as a binary variable: consistent users were participants who reported always using condoms during anal intercourse, whereas inconsistent users did not.

Results from the few studies conducted on condom use among MSM and transgender women in Southeast Asia are consistent with the global findings. Having a higher perceived risk of HIV was associated with inconsistent condom use among MSM and transgender women in Thailand [17]. MSM and transgender women in Indonesia who reached a higher educational level, had fewer depressive symptoms in the past week, and had no history of abuse as a child were more likely to report consistent condom use [18]. Drug use and commercial sex decreased the likelihood of condom use [18]. These studies, however, did not differentiate factors based on type of sexual partners (commercial vs. non-commercial). They also mostly include both transgender women and MSM, not focusing on transgender women specifically.

Cambodia is an important context in which to explore factors associated with condom use among transgender women. The prevalence of HIV among transgender women in Cambodia is between 4.6 to 5.9% [11, 16]. This is double the rate among MSM (2.3%) [25], and about 20 times that of the general adult population (0.3%) [26]. Transgender women in Cambodia also report inconsistent condom use more frequently than MSM, have engaged in sex work at higher rates, and admit to sexual intercourse with both men and women [16, 27]. Despite their higher risk profile and unique characteristics, until 2012, transgender women were not recognized as a separate group from MSM [16]. Therefore, public health interventions targeted specifically at transgender women are less developed than those for other key populations [28, 29].

In this population, consistent condom use with commercial and non-commercial partners varies widely. Weissman et al. (2016) found that 84% of participants used a condom during last sex, whereas Yi et al. (2017) reported 62% [11, 16]. Unlike other studies that show decreased condom use among non-commercial partners, in Weissman et al. (2016), consistent condom use among commercial and non-commercial male partners in the last six months was equal, at around 45% [16]. On the other hand, participants surveyed in Yi et al. (2017) were more likely to consistently use condoms with commercial partners in the last three months [11].

Like these two studies, most research focuses on condom use generally, or specifically with commercial partners [4, 5, 16]. There is a paucity of research on factors associated with condom use in non-commercial relationships. Past studies identified that an important gap in the literature is to understand condom use based on partner types and sexual acts [16]. This study was therefore conducted to explore factors associated with consistent condom use among sexually-active transgender women in Cambodia, specifically with their non-commercial partners.

## Methods

### Study sites and participants

Data used for this study were collected as part of a National Integrated Biological and Behavioral Survey conducted between December 2015 and February 2016 in the capital city of Phnom Penh and other 12 provinces of Cambodia as a partnership between the National Center for HIV/AIDS, Dermatology and STD (NCHADS) and a consortium of HIV/AIDS Flagship Project (KHANA, FHI360, PSI). The 13 city and provinces selected are high priority sites for HIV intervention programs, as they have a large population size of transgender women and higher burden of HIV. The Respondent Driven Sampling (RDS) method was used to recruit study participants, and the Strengthening the Reporting of Observational Studies in Epidemiology for RDS Studies (STROBE-RDS) statement was used as a guide for this study [30].

Transgender women were recruited in six locations in Phnom Penh and 14 locations in the remaining 12 provinces. The number of locations was determined based on the proportion of the required sample size and estimated population size of transgender women in each site. Individuals would be included in the study if they were: (1) aged 18 years or older, (2) biologically male at birth and currently self-identified as female, (3) reporting having sexual intercourse with at least one man in the past 12 months, (4) willing to participate in the study and able to provide a written consent, and (5) able to communicate in Khmer, the national language of Cambodia.

The initial participants (seeds) were recruited at drop-in centers, community centers, private houses, or offices of community-based NGOs convenient to the participants. Initially, an outreach worker working at each study site selected four seeds (two for the age group of 18–24 and the other two for the age group of 25 or older). To be selected as a seed, an individual must meet the eligibility criteria determined by using an eligibility screening tool and have a well-established social network knowing at least 10 transgender women within their given location. Three coupons were provided to each eligible seed to refer three transgender women. For a successful referral, seeds would receive an incentive of US$2. They were expected to reach three to six recruitment waves in each site. The data collection team would recruit additional seeds based on the above criteria if the enrollment was halted because the initial seeds did not recruit participants or all recruitment chains had dried up (i.e. stopped recruiting).

### Training and data collection

All research team members attended a three-day data collection training that covered a review of the study protocol, confidentiality and privacy protection measures, and interview techniques. The team had an opportunity to rehearse study procedures and questionnaire administration through a pretest. Data collection team leaders conducted regular daily review sessions with interviewers to review progress and communicate any problems that may emerge during the data collection.

Two data collection teams were formed with eight members each; they included one field supervisor, one counselor, one lab technician, and five interviewers. Participant eligibility screening was performed by the field supervisor of each team. Each consenting participant was assigned a unique personal identification number that was used to link all data collected from each participant, but the number was not linked to any participant personal identifiers. Prior to data collection, each participant was requested to provide a written informed consent that they understood the study procedures, potential risks and benefits, and their rights to decline or withdraw themselves from the study. The participant was then interviewed in a private room using an Android tablet. Each participant received US$4 in cash for their time and transport compensation with a package of three condoms.

### Questionnaire development

The questionnaire was initially developed in English, translated into Khmer, and back-translated into English. Important stakeholders working on HIV key populations and representatives of transgender women communities in Cambodia were consulted to validate the questionnaire. The questionnaire was pretested with 20 transgender women in Phnom Penh, who were later excluded from the main study, as part of the data collection training to ensure that wording and contents of the questionnaire were culturally acceptable and clearly understood by the participants.

The questionnaire was constructed based on items adapted from recent community-based surveys among HIV key populations in Cambodia [11, 16, 31, 32] and the most recent Cambodia Demographic and Health Survey [33]. Socio-demographic characteristics of the participants included age, study sites (urban, rural), marital status, main occupation, completed years of formal education, average monthly income in the past six months, duration living in the current city, perceived family economic status, and gender identity. Participants were also asked about gender expression and utilization of gender-affirming hormones and surgeries.

HIV risks included participants’ sexual behaviors and condom use with different types of partners in the past three months. Participants’ substance use behaviors were also assessed, including alcohol and different kinds of illicit drugs (amphetamine-type stimulants, heroin, marijuana, etc.). For STIs, participants were asked about their experiences of several symptoms in the past three months such as cuts or sores, swelling in the genital area, abnormal urethral discharge, symptoms on the anus, and symptoms in the mouth/throat as well as care seeking behaviors for the most recent symptoms.

To measure access to community-based HIV programs, participants were asked whether they had received any of the following services from a community-based organization in the past six months: (1) condom and lubricant distribution, (2) screening for HIV and STIs, (3) HIV and sexual reproductive health education, (4) other health services such as HIV and STI treatment, (5) legal aid services, and (6) online services developed for transgender women (websites, Facebook pages, hotline calls, etc.).

### Data analyses

Stata (StataCorp LP, version 14.1) was used for data analyses. The RDS-adjusted percentages with 95% CI were calculated using Stata’s “rds_network,” “rds,” and “bootstrap_b: rds,” which was adapted from www.respondentdrivensampling.org. Since RDS technique does not work with continuous variables, all continuous variables were converted into categorical variables. Individual weights based on the outcome variables were created using RDS’ Stata command and used in both bivariate and multivariate analyses. Weighted bivariate analyses were conducted using χ^2^ test (or Fisher’s exact test when a cell count was smaller than five) to compare the proportion of consistent condom users by socio-demographic characteristics, substance use, HIV risks, and access to community-based HIV programs.

A weighted multivariate logistic regression model was constructed to explore independent factors associated with consistent condom use, defined as always using condoms with male non-commercial partners (partners with whom the sexual acts were not in exchange for money or gifts) in the past three months. Variables associated with consistent condom use in bivariate analyses at a level of *p-*value < 0.25 were included simultaneously in the model. Adjusted odds ratio (AOR) were obtained and presented with a CI and *p*-value.

### Ethical considerations

This study was approved by the National Ethics Committee for Health Research (NECHR) of Ministry of Health in Cambodia (No. 420 NECHR) and FHI 360’s Protection of Human Subjects Committee (PHSC No. 713897). A written informed consent was obtained from each participant. Privacy and confidentiality of participants were strictly protected by conducting the interviews in a private room and removing all personal identifiers from the study documents.

## Results

### Respondent driven sampling (RDS)

**Fig. 1:**
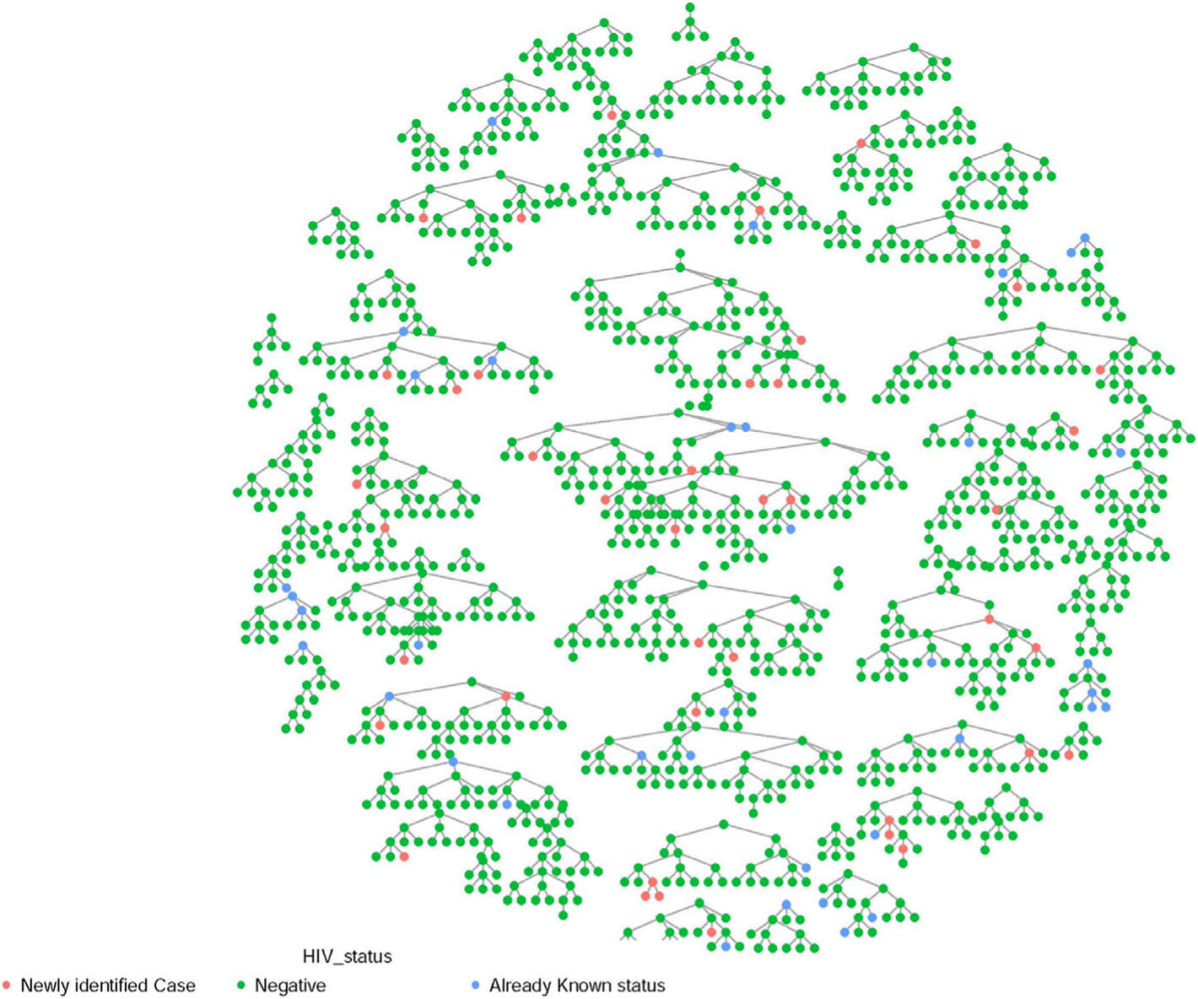
Overview of Respondent Driven Sampling recruitment of the study participants. Notes: One node represents a transgender woman participant. Each line connecting node represents one ‘wave’ of recruitment. Of each network, the top node represents the initial seed

Figure 1 shows the overview of RDS recruitment of transgender women in the national survey. In this Figure, there are 1375 nodes, representing 1375 transgender women included in the survey, derived from 80 initial seeds plus 1295 recruits. Of these 80 seeds, 76 had referred at least one of their peers. During the recruitment, 3267 coupons were distributed (three coupons per initial seed and recruiter), and a total of 1371 coupons were returned, giving the coupon return rate of 42.0%. Of people who returned with the coupons, 1295 (94.0%) participated in the study. The longest wave of the recruitment was 10, with a median wave of three [interquartile range (IQR) 2–5]. The median number of ‘personal networks’ (the number of transgender women each participant had a social relationship with) was 10 (IQR 5–20). Of the successful 76 seeds above, the median number of recruited participants per initial seed was 11 (IQR 5–25).

### Characteristics of participants

This study included 1202 transgender women who reported having anal intercourse with at least one male non-commercial partner in the past three months with a mean age of 26.0 (SD = 7.0). Of the total, 41.5% reported always using condoms in anal intercourse with male non-commercial partners in the past three months, classified as consistent condom users. Reasons for not using condoms included being in a relationship (41.6%), partner not being infected with HIV or an STI (18.5%), being under drug influence (3.2%), condoms being unavailable (17.0%), better sensation during sex without condoms (13.5%), and partner’s refusal (10.7%). Sources of condoms included outreach workers of an NGO (73.1%), pharmacy (45.6%), condom sale representative (15.7%), hotels/guesthouses (10.1%), grocery stores (12.1%), minimarts (9.2%), condom outlets (2.3%), or other sources (2.7%).

As shown in Tables 1, 83.0% of the participants were residing in an urban community. More than two-thirds (78.1%) were never married and not living with a partner, and 46.0% had attained at least 10 years of formal education. The proportion of consistent condom users was significantly higher among participants living in urban communities and participants who had attained at least 10 years of formal education.

**Table 1:** Characteristics of transgender women who reported always and not always using condoms with male non-commercial partners (*n* = 1202)

Socio-economic characteristics			Condom use with male non-commercial partner in the past 3 months		
	Crude *n* (%)	RDS-adjusted % (95% CI)	RDS-adjusted % (always)	RDS-adjusted % (not always)	P-value^*^
Age group					
18–24	620 (51.6)	57.7 (53.0, 62.4)	56.0	56.2	0.16
25–34	457 (38.0)	33.5 (29.6, 37.4)	31.8	35.8	
35 or older	125 (10.4)	8.8 (6.0, 11.6)	12.3	8.0	
Living in urban community	998 (83.0)	78.8 (72.0, 85.5)	82.9	72.9	0.01
Marital status					0.42
Married	5 (0.4)	0.4 (0.2, 0.7)	0.2	0.5	
Divorced/widowed	16 (1.3)	1.5 (0.9, 2.1)	1.2	1.7	
Never married	1170 (97.3)	97.2 (93.1, 99.2)	98.2	96.5	
Missing	11 (0.9)	1.0 (0.7, 1.3)	0.4	1.4	
Formal education attained (in year)					
0–6	268 (22.3)	24.7 (20.8, 28.7)	25.8	23.1	0.004
7–9	381 (31.7)	33.3 (29.5, 37.1)	25.2	37.6	
10 or more	553 (46.0)	42.0 (38.1, 45.9)	49.0	39.3	
Main occupation					
Unemployed	56 (4.7)	5.6 (3.8, 7.4)	5.0	4.9	0.08
Hairdresser/beautician	425 (35.4)	31.4 (26.9, 36.0)	28.7	32.0	
Office worker	76 (6.3)	5.7 (3.4, 7.9)	7.5	4.7	
Labor/farmer	220 (18.3)	22.1 (17.7, 26.6)	16.9	24.5	
Self-employed	130 (10.8)	10.3 (7.0, 13.5)	12.0	11.2	
Entertainment worker	171 (14.2)	12.9 (9.3, 16.4)	13.6	12.4	
Student	87 (7.2)	9.3 (6.0, 12.7)	49.4	50.6	
Other	37 (3.1)	2.7 (1.7, 3.7)	11.8	8.4	
Monthly income (in US$)					
< 100	297 (24.7)	27.9 (24.2, 31.7)	29.0	26.8	0.38
100–199	465 (38.7)	38.7 (34.9, 42.5)	35.0	41.4	
200–299	239 (19.9)	18.3 (14.4, 22.2)	20.0	17.0	
> 300	201 (16.7)	15.1 (12.7, 17.5)	16.1	14.8	
Always dressed as a woman	620 (51.6)	58.1 (53.5, 62.8)	41.5	44.8	0.38

*Abbreviations:* *CI* confidence interval, *RDS* respondent driven sampling

^*^ *Chi-square test (or Fisher’s exact test when a cell count was smaller than 5) was used*

### Substance use

Table 2 shows that 22.3% of the total participants reported not drinking any kind of alcohol, and 29.0% reported drinking alcohol two times or more per week in the past three months. Of the drinkers, 25.7% reported drinking five or more cans of beer or glasses of wine on a typical day. Regarding illicit drug use, 10.3% reported using some kinds of amphetamine-type stimulants (ATS), 0.8% using other drugs such as heroin/opium or marijuana in the past three months, and 10% using drugs before or while having sex. The proportion of consistent condom users was significantly higher among participants who reported using alcohol four times or more per week, using an illicit drug, and using drugs before or while having sex in the past three months.

**Table 2:** Substance use among transgender women who reported always and not always using condoms with male non-commercial partners (*n* = 1202)

Substance use			Condom use with male non-commercial partner in the past 3 months		
	Crude *n* (%)	RDS-adjusted % (95% CI)	RDS-adjusted % (always)	RDS-adjusted % (not always)	P-value^*^
Frequency of alcohol drinking in the past 3 months					
Never	293 (24.4)	22.3 (18.9, 25.6)	19.3	26.9	0.003
≤Once a month	291 (24.2)	26.8 (23.5, 30.1)	26.3	26.6	
2–4 times per month	267 (22.2)	22.0 (18.8, 25.2)	20.9	22.8	
2–3 times per week	168 (14.0)	13.8 (10.8, 16.8)	15.1	12.5	
≥4 times per week	183 (15.2)	15.2 (11.9, 18.4)	18.5	11.2	
Type of illicit drug most commonly used in the past 3 months					
Never used	1068 (88.9)	88.8 (85.7, 91.9)	84.2	94.1	< 0.001
ATS (amphetamine, methamphetamine)	124 (10.3)	10.6 (7.4, 13.8)	14.9	15.9	
Other drugs (heroin/opium/marijuana)	10 (0.8)	0.6 (0.0, 1.3)	1.0	0.1	
Used drugs before or while having sex	77 (6.4)	6.8 (4.2,9.5)	8.9	3.8	0.01

*Abbreviations:* *ATS* amphetamine-type stimulants, *CI* confidence interval, *RDS* respondent driven sampling

^*^ *Chi-square test (or Fisher’s exact test when a cell count was smaller than 5) was used*

### HIV risk behaviors

As shown in Tables 3, 85.7% reported receptive roles in anal intercourse with men in the past three months. More than one-third (34.0%) reported having anal sex with men in exchange for money or gifts in the past three months, with 22.5% having ≥2 male commercial partners and 17.6% always using condoms with this type of partners in the past three months. More than two-thirds (*n* = 821, 68.4%) perceived that they were likely or very likely to be HIV infected; of whom, 15 (1.8%) reported that they were living with HIV, and 73.3% (*n* = 11) of those living with HIV were on ART. Less than half (42.9%) reported having been tested for HIV in the past six months, and 14.7% having an STI symptom in the past three months. The proportion of consistent condom users was significantly higher among participants who reported not always using condoms with male commercial partners in the past three months, reported having been tested for HIV in the past six months, and perceived that they are likely to be HIV infected.

**Table 3:** HIV risks among transgender women who reported always and not always using condoms with male non-commercial partners (*n* = 1202)

HIV risks			Condom use with male non-commercial partner in the past three months		
	Crude *n* (%)	RDS-adjusted % (95% CI)	RDS-adjusted % (always)	RDS-adjusted % (not always)	*P*-value^*^
Main role in anal sex with men in the past 12 months					
Insertive	26 (2.2)	2.0 (1.0, 2.9)	2.9	1.3	0.32
Receptive	1043 (86.8)	85.7 (82.8, 88.6)	84.9	85.9	
Both	133 (11.1)	12.3 (9.5, 15.1)	12.3	12.7	
Had sex with male commercial partners in the past 3 months	444 (36.9)	34.0 (29.6, 38.3)	33.3	36.3	0.43
Had ≥2 male commercial partners in the past 3 months	310 (25.8)	22.5 (19.1, 25.9)	21.5	24.3	0.42
Always used condoms with male commercial partners	243 (20.2)	17.6 (14.1, 21.0)	14.1	24.9	< 0.001
Had an STI symptom in the past 12 months	175 (14.6)	14.7 (11.5, 17.8)	11.3	17.6	0.005
Tested for HIV in the past 6 months	617 (51.3)	42.9 (37.9, 47.8)	51.6	43.8	0.04
Perceived likelihood of HIV infection					
Very Likely	128 (10.7)	7.0 (5.4, 8.6)	6.2	9.8	< 0.001
Likely	693 (57.7)	58.0 (54.0, 62.0)	48.5	63.3	
Unlikely	293 (24.4)	27.1 (23.4, 30.8)	33.8	19.8	
Very Unlikely	88 (7.3)	7.9 (5.9, 10.0)	9.9	5.3	

*Abbreviations:* *CI* confidence interval, *HIV* human immunodeficiency virus, *RDS* respondent driven sampling, *STI* sexually transmitted infections

^*^ *Chi-square test (or Fisher’s exact test when a cell count was smaller than 5) was used*

### Access to community-based HIV services

Of the total, 35.2% had been reached by at least one form of HIV services provided by community-based NGOs. The major services they had received included condom and lubricant distribution (39.4 and 28.2%, respectively), HIV education (36.5%), and general online HIV services (34.9%). The proportion of consistent condom users was significantly lower among participants who had been reached by community-based HIV services including HIV education, condom and lubricant distribution, HIV/syphilis testing, and different kinds of online services (see Table 4).

**Table 4:** Access to community-based HIV services among transgender women who reported always and not always using condoms with male non-commercial partners (*n* = 1202)

Community-based HIV services received in the past 6 months			Condom use with male non-commercial partner in the past 3 months		
	Crude *n* (%)	RDS-adjusted % (95% CI)	RDS-adjusted % (always)	RDS-adjusted % (not always)	P-value^*^
Reached by community-based HIV services	552 (45.9)	35.2 (30.7, 39.7)	33.8	45.4	0.001
Type of HIV services received					
HIV education	439 (36.5)	27.6 (24.2, 31.1)	28.1	32.6	0.19
Condom distribution	474 (39.4)	29.3 (26.0, 32.6)	26.1	40.5	< 0.001
Lubricant distribution	339 (28.2)	20.8 (17.5, 24.2)	18.9	29.8	< 0.001
HIV/syphilis testing	339 (28.2)	21.9 (18.4, 25.4)	19.0	31.5	< 0.001
Legal support services	46 (3.8)	2.6 (1.4, 3.7)	2.1	1.7	0.17
General online HIV services	419 (34.9)	62.3 (58.0, 66.5)	63.5	62.8	0.81
Visited MStyle Facebook pages (for MSM)	114 (9.5)	5.8 (4.3, 7.3)	6.8	7.8	0.60
Visited MStyle websites (for MSM)	75 (6.2)	3.6 (2.4, 4.8)	3.5	5.8	0.11
Visited Facebook pages (for transgender women)	128 (10.7)	6.3 (4.4, 8.2)	7.2	9.1	0.31
Visited websites (for transgender women)	84 (7.0)	3.8 (2.3, 5.3)	3.3	7.6	< 0.001
Received Voice messages from Voice4U about HIV	72 (6.0)	4.7 (3.0, 6.4)	3.7	7.5	0.02
Called Voice4U for HIV information	69 (5.7)	3.8 (2.5, 5.2)	3.0	6.8	0.01

Notes: *CI* confidence interval, *HIV* human immunodeficiency virus, *MSM* men who have sex with men, *RDS* respondent driven sampling, *STI* sexually transmitted infections

^*^ *Chi-square test (or Fisher’s exact test when a cell count was smaller than 5) was used*

### Factors associated with consistent condom use

Results of the multivariable logistic regression analysis are shown in Table 5. After adjustment, the likelihood of consistent condom use was significantly higher among participants who resided in urban communities (AOR = 1.7, 95% CI = 1.1–2.6), had attained at least 10 years of formal education (AOR = 1.8, 95% CI = 1.2–2.7), perceived that they were likely or very likely to be HIV infected (AOR = 2.9, 95% CI = 2.0–4.1), reported drinking alcohol two to three times per week in the past three months (AOR = 3.1, 95% CI = 1.1–8.3), reported using ATS (AOR = 1.9, 95% = 1.1–3.8) or other drugs (AOR = 7.6, 95% CI = 1.5–39.5) in the past three months, and reported not always using condoms with male commercial partners in the past three months (AOR = 4.3, 95% CI = 1.8–10.4) compared to that among their respective reference group. The likelihood of consistent condom use was significantly lower among participants who reported having been reached by community-based HIV services in the past three months (AOR = 0.6, 95% CI = 0.4–0.8) and having visited websites developed specifically for transgender women in the past six months (AOR = O.4, 95% CI = 0.2–0.7) compared to their respective reference group.

**Table 5:** Factors associated with consistent condom use with non-commercial partners among transgender women in multiple logistic regression model (*n* = 1202)

Independent variables	Consistent condom use with non-commercial partner in the past 3 months	
	AOR (95% CI)	*p*-value^*^
Community type		
Rural	Reference	
Urban	1.7 (1.1, 2.6)	0.02
Formal education attained (in year)		
0–6	Reference	
7–9	1.2 (0.6, 1.6)	0.98
≥10	1.8 (1.2, 2.7)	0.003
Perceived likelihood of HIV infection		
Unlikely/very unlikely	Reference	
Likely/very likely	2.9 (2.0, 4.1)	< 0.001
Frequency of alcohol drinking in the past 3 months		
No	Reference	
Yes	0.6 (0.4, 0.8)	0.002
Visited websites developed specifically for transgender women in the past 6 months		
No	Reference	
Yes	0.4 (0.2, 0.7)	0.004

Notes: *AOR* adjusted odds ratio, *ATS* amphetamine-type stimulants, *CI* confidence interval

^*^ *Age, marital status, education level, income, and variables associated with HIV infection in the bivariate analyses at a level of p < 0.05 were simultaneously included in the model*

## Discussion

In this study, 41.5% of participants reported always using condoms in anal intercourse with male non-commercial partners in the past three months. The two previous studies of Cambodian transgender women’s sexual behaviors, which also used RDS method, reported on condom use with commercial and non-commercial partners. Weissman et al. (2016) found that condom use was about the same—around 45%—with both kinds of partners over the previous six months [16]. On the other hand, participants surveyed in Yi et al. (2017) were more likely to consistently use condoms with commercial partners in the past three months (60% vs. 38% with non-commercial) [11]. These variations could indicate that some transgender women are better able to negotiate condom use with commercial partners. There are also structural elements to condom use within sex work: some employers may provide, or even require condoms, and some may not. Since these studies were conducted in different provinces, the location of the sex work (venue-based, street-based, etc.) could influence condom use.

Regardless of location, higher consistent condom use with commercial partners relative to non-commercial partners is consistent with the overall literature [12, 13, 18, 34]. The reasons for differential condom use by partner have been explained in multiple ways in other studies—because of discrimination they have faced, transgender women may want to affirm their gender identity, and their partner’s desire for them by having condomless intercourse [13, 34]. Condoms can also undermine the feelings of intimacy with romantic partners, a factor not unique to transgender relationships [12]. We also found that participants were more likely to use condoms with non-commercial partners if they did not always use condoms with commercial partners. This introduces an element of risk calculation into the condom use decision-making. If transgender women use condoms with commercial partners, they may feel that they are protected from HIV and other STIs, so they are freer to engage in condomless intercourse with their non-commercial partners. This interpretation is consistent with our findings related to perceived HIV risk. Although these studies mostly recruited participants through word-of-mouth outreach, they did not employ RDS analyses to correct for the non-random sample. Therefore, their conclusions must be interpreted as not fully comparable with our RDS-adjusted estimates.

In this study, we found a dose-response effect in the relationship between self-perceived HIV risk and condom use with non-commercial partners. The lower the participant’s perceived risk of HIV, the lower the odds of consistent condom use with non-commercial partners. This means that our participants’ condom use with non-commercial partners matched their risk perception. A 2017 study in Thailand found the opposite—that increased HIV risk perception was associated with inconsistent condom use [17]. The Thai data reflected that the participants may not have taken their perceived HIV risk into account when practicing condomless intercourse. On the other hand, our participants could assess their HIV risk more accurately and act accordingly. However, this is only perceived risk—transgender women in our sample could be assuming that, in engaging in condomless intercourse with their non-commercial partners, they are safe from HIV and other STIs.

Although not all of the results were statistically significant, we found a trend that the more alcohol consumption within the past three months, the higher the odds of consistent condom use with non-commercial partners. This finding is inconsistent with numerous other studies that demonstrated a decreased likelihood of condom use with drug and alcohol use [6, 12, 18]. We assessed alcohol intake by asking the number of days they drank on a given week. We did not specifically ask participants when they drank alcohol, however, so the alcohol consumption could have been during sexual encounters with commercial partners, or unrelated to sexual intercourse in general. Future studies should employ more accurate alcohol measures that take into account alcohol dependence or problematic alcohol use.

Finally, we found that participants who had been reached by community-based HIV services in the past three months had a lower odds of consistent condom use with non-commercial partners, as compared to participants who did not receive the services. This may reflect a failure of community-based HIV programs to teach HIV prevention behaviors adequately. However, there is not enough data to jump to this conclusion. Perhaps the participants who were not reached by the programs were not very sexually active or engaged less in sexual risk behaviors such as multiple sexual partners, for instance. Moreover, some service seeking behaviors, such as HIV testing or searching for online HIV services may indicate a higher risk profile, which is consistent with a lower rate of consistent condom use.

Some limitations of the study should be noted. This study included only the capital city and 12 provinces with a large population size of transgender women and higher burden of HIV. These city and provinces are prioritized sites for HIV intervention programs. Therefore, findings from this study may not be generalized at a national level. Specific to our RDS methodology, the initial seeds of participants were identified and recruited by outreach workers working for community-based NGOs, which could be biased towards transgender women who are participants in their programs and therefore more educated on sexual health. In addition, participants were more likely to be more socially connected than non-participants and were more likely to recruit people who were like themselves, and therefore, non-independence may be an issue. However, we have tried to account for some of these limitations by accounting for networks in our analyses. Furthermore, with RDS, after a certain number of waves, the characteristics of the participants would become independent from those of the seeds. Another limitation is the self-reporting measures on sensitive issues such as sexual behaviors and substance abuse that may have encountered over- and under-reporting biases. Finally, the monetary incentive given to the participants to recruit seeds may have affected their genuine motivation to partake in the study to certain extent and influence their responses. Nevertheless, sufficient due measures were taken in data collection procedures to minimize these potential effects.

## Conclusions

This study confirms the persistently low rates of condom use, particularly in non-commercial relationship among transgender women in Cambodia. It showed that lower perceived risk of HIV was associated with lower condom use rates, regardless of the actual risk. This is confirmed by the relationship between consistent condom use with commercial vs. non-commercial sexual partners. Participants were more likely to consistently use condoms with non-commercial partners if they inconsistently used condoms with commercial partners. This risk assessment behavior may have exacerbated vulnerability to HIV and other STIs and explain the increase of HIV prevalence among this population, despite the substantial reduction of new HIV infections in the general population. However, this study demonstrates the complexity of the factors that influence condom use with non-commercial partners among this population, including alcohol and drug use, and use of various health services. To address these concerns, efforts towards education about harmful effects of multiple, concurrent relationships, and inconsistent condom should be reinforced among transgender women.

## Abbreviations

AIDS: Acquired immune deficiency syndrome
AOR: Adjusted odd ratio
CI: Confidence interval
HIV: Human immunodeficiency virus
IQR: Interquartile range
MSM: Men who have sex with men
NCHADS: National Center for HIV/AIDS, Dermatology and STD
NECHR: National Ethics Committee for Health Research
NGO: Non-governmental organization
RDS: Respondent driven sampling
SD: Standard deviation
STI: Sexually transmitted infection
USD: United States dollar

## References

[1] Guadamuz TE, Wimonsate W, Varangrat A, Phanuphak P, Jommaroeng R, McNicholl JM (2011). HIV prevalence, risk behavior, hormone use and surgical history among transgender persons in Thailand. AIDS Behav.

[2] Silva-Santisteban A, Raymond HF, Salazar X, Villayzan J, Leon S, McFarland W (2012). Understanding the HIV/AIDS epidemic in transgender women of Lima, Peru: results from a sero-epidemiologic study using respondent driven sampling. AIDS Behav.

[3] Brennan J, Kuhns LM, Johnson AK, Belzer M, Wilson EC, Garofalo R (2012). Syndemic theory and HIV-related risk among young transgender women: the role of multiple, co-occurring health problems and social marginalization. Am J Public Health.

[4] Chakrapani V, Newman PA, Shunmugam M, Logie CH, Samuel M (2017). Syndemics of depression, alcohol use, and victimisation, and their association with HIV-related sexual risk among men who have sex with men and transgender women in India. Glob Public Health.

[5] Nemoto T, Iwamoto M, Perngparn U, Areesantichai C, Kamitani E, Sakata M (2012). HIV-related risk behaviors among kathoey (male-to-female transgender) sex workers in Bangkok, Thailand. AIDS Care.

[6] Operario D, Nemoto T, Iwamoto M, Moore T (2011). Unprotected sexual behavior and HIV risk in the context of primary partnerships for transgender women. AIDS Behav.

[7] Baral SD, Poteat T, Strömdahl S, Wirtz AL, Guadamuz TE, Beyrer C (2013). Worldwide burden of HIV in transgender women: a systematic review and meta-analysis. Lancet Infect Dis.

[8] Chhim S, Ngin C, Chhoun P, Tuot S, Ly C, Mun P (2017). HIV prevalence and factors associated with HIV infection among transgender women in Cambodia: results from a national integrated biological and behavioral survey. BMJ Open.

[9] De Santis JP (2009). HIV infection risk factors among male-to-female transgender persons: a review of the literature. J Assoc Nurses AIDS Care..

[10] Nemoto T, Cruz T, Iwamoto M, Trocki K, Perngparn U, Areesantichai C (2016). Examining the sociocultural context of HIV-related risk behaviors among Kathoey (male-to-female transgender women) sex Workers in Bangkok, Thailand. J Assoc Nurses AIDS Care.

[11] Yi S, Ngin C, Tuot S, Chhoun P, Chhim S, Pal K (2017). HIV prevalence, risky behaviors, and discrimination experiences among transgender women in Cambodia: descriptive findings from a national integrated biological and behavioral survey. BMC Int Health Hum Rights.

[12] Nemoto T, Operario D, Keatley J, Han L, Soma T (2004). HIV risk behaviors among male-to-female transgender persons of color in San Francisco. Am J Public Health.

[13] Melendez RM, Pinto R (2007). It’s really a hard life’: love, gender and HIV risk among male-to-female transgender persons. Cult Health Sex.

[14] Beyrer C, Baral SD, van Griensven F, Goodreau SM, Chariyalertsak S, Wirtz AL (2012). Global epidemiology of HIV infection in men who have sex with men. Lancet..

[15] Baggaley RF, White RG, Boily M-C (2010). HIV transmission risk through anal intercourse: systematic review, meta-analysis and implications for HIV prevention. Int J Epidemiol.

[16] Weissman A, Ngak S, Srean C, Sansothy N, Mills S, Ferradini L (2016). HIV prevalence and risks associated with HIV infection among transgender individuals in Cambodia. PLoS One.

[17] Plotzker R, Seekaew P, Jantarapakde J, Pengnonyang S, Trachunthong D, Linjongrat D (2017). Importance of risk perception: predictors of PrEP acceptance among Thai MSM and TG women at a community-based health service. J Acquir Immune Defic Syndr.

[18] Safika I, Johnson TP, Cho YI, Praptoraharjo I (2014). Condom use among men who have sex with men and male-to-female Transgenders in Jakarta, Indonesia. Am J Mens Health.

[19] Ramirez-Valles J, Garcia D, Campbell RT, Diaz RM, Heckathorn DD (2008). HIV infection, sexual risk behavior, and substance use among Latino gay and bisexual men and transgender persons. Am J Public Health.

[20] Clements-Nolle K, Guzman R, Harris SG (2008). Sex trade in a male-to-female transgender population: psychosocial correlates of inconsistent condom use. Sex Health.

[21] Nuttbrock L, Bockting W, Rosenblum A, Hwahng S, Mason M, Macri M (2013). Gender abuse, depressive symptoms, and HIV and other sexually transmitted infections among male-to-female transgender persons: a three-year prospective study. Am J Public Health.

[22] Kosenko KA (2011). Contextual influences on sexual risk-taking in the transgender community. J Sex Res.

[23] Lombardi EL, Wilchins RA, Priesing D, Malouf D (2002). Gender violence: transgender experiences with violence and discrimination. J Homosex.

[24] Wilson EC, Garofalo R, Harris DR, Belzer M (2010). Sexual risk taking among transgender male-to-female youths with different partner types. Am J Public Health.

[25] Yi S, Chhim S, Chhoun P, Tuot S, Ly C, Mun P (2016). Men who have sex with men in Cambodia: population size, HIV risky behaviors, and HIV prevalence. Am J Epidemiol Infect Dis.

[26] Mun P, Heng S, Tuot S, Morgan P, Pal K, Chhoun P (2016). National HIV sentinel survey among women attending antenatal care clinics in Cambodia in 2014.

[27] Chhorvann C, Liu K-L (2007). Behavioral Surveillance Surve: HIV/AIDS Related Sexual Behaviors Among Sentinel Groups.

[28] Liu K-L, Chhorvann C (2012). The BROS KHMER: Behavioral Risks On-Site Serosurvey among At-Risk Urban Men in Cambodia. Phnom Penh, Cambodia; 2010.

[29] Chhorvann C, Vonthanak S (2011). Estimations and Projections of HIV/AIDS in Cambodia 2010–2015.

[30] White RG, Hakim AJ, Salganik MJ, Spiller MW, Johnston LG, Kerr L (2015). Strengthening the reporting of observational studies in epidemiology for respondent-driven sampling studies: "STROBE-RDS" statement. J Clin Epidemiol.

[31] Yi S, Tuot S, Chhoun P, Brody C, Tith K, Oum S (2015). The impact of a community-based HIV and sexual reproductive health program on sexual and healthcare-seeking behaviors of female entertainment workers in Cambodia. BMC Infect Dis.

[32] Yi S, Tuot S, Chhoun P, Pal K, Ngin C, Choub SC (2016). Improving prevention and care for HIV and sexually transmitted infections among men who have sex with men in Cambodia: the sustainable action against HIV and AIDS in communities (SAHACOM). BMC Health Serv Res.

[33] National Institute of Statistics (2014). Directorate general for health, and ORC macro. Cambodia demographic and health survey 2014.

[34] Bockting WO, Robinson BE, Rosser BR (1998). Transgender HIV prevention: a qualitative needs assessment. AIDS Care.

